# Cases Illustrating the Pathology of What Are Called “Cold Cases” of Bilious Fever

**Published:** 1830

**Authors:** James C. Finley


**Art. VII.**—*Cases illustrating the pathology of what are called "Cold cases" of Bilious Fever, with remarks by* James C. Finley, M. D.

So much has been written upon the subject of bilious fever, that it is with reluctance I again appear before the publick in connection with this subject. Having published, however, an essay on the "Bilious Fever of Georgia," in a former number of this Journal, I hope that a few cases, in illustration of the pathology and treatment advanced in that essay, will not be unacceptable to our readers.

Case 1. Mrs. W. aged about 50, after a very protracted case of bilious was confined with an attack of Intermittent fever, on the 1st October, 1826. On the next day I saw her, and was informed that the chill on the preceding day had commenced about 10 in the morning, and continued about four hours. At the expiration of this time, her attendants permitted her to satisfy her urgent thirst by a copious draught of cold water, and in a short time, the cold stage terminated by a free perspiration without the intervention of any apparent reaction. I found her in a state of great debility, the powers of the circulation very much impaired, complaining of chilliness, with the surface of the body pale and contracted. The surface of the body was directed to be rubbed with Tinct. of capsicum, the carb. of ammonia was given internally, and although she stated calomel had generally operated very unkindly upon her system, I ventured to give it, in combination with pills of Rhubarb and Aloes, at intervals, so that she might take 5 grs. in the course of the day.

At 3 o'clock on the next morning, I again saw her. The fever had returned on the preceding evening, with great anxiety, restlessness and incessant efforts to vomit. The stomach was relieved by a sinapism, and by draughts of the carb. ammo., and a teaspoonful of ladanum was prescribed at eight o'clock, in anticipation of the chill. The carbonate of ammonia to be taken through the day if the symptoms should require it.

In the evening had rested comfortably during the day, without chill or fever. Directed 25 gtts. of ladanum to be taken with 1-2 oz. castor oil.

In the morning the oil had operated; patient clear of fever and gastric distress. Quinine 2 grs. every 2 hours.

In the evening, fever, gastric oppression, tenderness over the region of the stomach. Directed 1-2 oz. castor oil, and a blister to the epigastrium. In the morning the oil had operated copiously, producing very great depression. I directed an anodyne which was not taken, and in the evening I found her in a state of the most alarming debility, her evacuations passing insensibly. A full anodyne with the liberal administration of diffusible stimulants restored her from this situation. On the next morning she was free from fever, and took the sulph. of quina with a very good effect. Her health was very slowly and gradually restored.

Case 2. The writer of these remarks, in a few weeks after his convalescence from a very protracted case of fever, was exposed during the day and until late in the night, by riding in the rain on a very cold day. On the next day, (October 8, 1826), he was seized with a chill followed by fever with stupor. A dose of calomel acted as an emectic-o—cathartic, evacuating a quantity of viscid yellow bile; and on the next morning, he rode through a damp cold air, several miles to see a friend that was dangerously ill. On the 10th, the chill returned without any striking symptoms of congestion or inflammation, without pain or oppression, and continued from 9 A. M. until 2 P. M., terminating, as in the former case, without manifest reaction, by a free perspiration; and leaving the system completely prostrated.

The fourth day of his illness passed with very little fever, and on the 5th, the chill was prevented by 50 gtts. of ladanum with other diffusible stimulants. On the morning of the 6th, Dr. Childers, a physician of great observation and experience who very kindly attended him during the remainder of his illness, considered him to be out of danger and prescribed the Sul. quinœ. As his bowels had been neglected since the first day of his illness, a few grs. of rhubarb were prescribed. The

Sul. of quinine produced so distressing a state of general excitement that it was discontinued, and the rhubarb by its operation prostrated the system so much that the pulse sunk, leaving the patient with too much irritation to bear the free administration of stimulants, and with too much debility to admit of evacuants. He remained in a state of insensibility until 2 o'clock on the morning of the eighth day. In the mean time, the strength was supported by the cautious administration of the carbonate of ammonia, blisters were applied to the extremities, and contrary to the expectation of every one, the system was aroused, and the senses suddenly acquired their powers of perception. The appetite soon returned, but the mind as well as the body, long remained in a state of infantile weakness, and the feelings, amusements and trains of thought were those of childhood.

There was some reason to doubt whether the depression on the sixth day of the disease arose from the operation of the medicine, or was the consequence of the returning paroxysm of fever. The former was the more obvious cause, but the great irritability which prevailed would rather discountenance such an opinion ; and in addition, it will be observed, that it continued through two complete paroxysms of the fever.*

These two cases we think, present several important peculiarities. 1. The great length and severity of the cold stage with so entire an absence of the usual symptoms of congestion. Of my own feelings during the chill I have still a distinct recollection. There was no pain or oppression—but a slight sensation of chilliness creeping over the body, with such a torpor of the perceptive and intellectual faculties, that although perfectly sensible of the almost certain approach of death, produced none of those feelings of anxiety which arise so naturally in the mind

* Those who have read the essay to which I allude may remember, that it was stated that the bilious fever in that section of country was uniformly of the double tertian type, consisting of two paroxysms the one on the odd days, commencing in the morning and manifesting a disposition to terminate in the evening of the same day with an intermission more or less perfect; the other commencing on the afternoon of the even day and continuing through the night, frequently until within a short time of the paroxysm of the next day.

on similar occasions. The faculties of the mind, although impaired and almost prostrated, still preserved their regular and harmonious action. 2. The absence of fever shews that the hot stage of intermittent fever is not essential to the sweating stage; and that febrile action, though no doubt, a salutary effort of nature to repair the inroads made upon the animal economy, is not essential to those critical discharges by which she relieves herself. 3. Another curious circumstance is, that in this state of entire prostration of the vital powers so much irritability should have existed, as to preclude the use of the sulp. qui. —This medicine producing in one case, symptoms of gastro-enteritis, and in the other, a most distressing feeling of general excitement that seemed to pervade every fibre of the body. 4. The train of symptoms appear to point out a cause of fever very different from local inflammation, and if this did occur, it may very justly be considered as the effect of the fever, or of the too early administration of tonics. Lastly, they place in a very striking light the errors of those pathologists, who see no other cause of debility in congestive fevers but visceral engorgement; on the contrary, congestion is attended with direct as well as indirect debility, and the former, if not first in importance is at least first in the order of succession.

*Case* 3. I was called, Nov. 6th 1828, to visit A. H. a girl about 9 years of age. After a slight indisposition of two days, having taken a chill on the third day about 8 o'clock, I found her at eleven, still under its influence. She was perfectly insensible, the pulse indistinct, irregular and fluttering, and the surface of the body pale and flaccid—the blood in some places having settled in the superficial veins so as to give the surface a purplish hue; the respiration somewhat hurried and laborious. Bottles filled with warm water were applied to the extremities and axillœ; 10 gtt. of the "black drop" were administered; the carbonate of ammonia and infusion of serpentaria were assiduously given every hour, and sinapisms of a large size were applied over the stomach and to the extremities, which were suffered to remain until some degree of permanent inflammation was produced. The action of the sinapisms produced some degree of excitement, and about 2 o'clock, symptoms indicative of approaching convulsions occurred which we supposed would terminate her existence, but contrary to our expectations, they proved the harbinger of a favorable change. In a short time a general glow appeared on the surface, the pulse became fuller and more distinct, followed, in the course of the afternoon, by a slight diffused perspiration; and with a pleasure superior to any I ever experienced except from a similar source, I saw the unfavorable symptoms successively vanish.

On the next morning, no fever. Sulp. quina, Dovers Powder and calomel were given during the morning. In the afternoon, castor oil, which operated producing serous evacuations only. Slight return of fever in the afternoon and through the night.

On the next day, the 5th of her illness, calomel 10 grs. and Dovers Powder 5 grs. were given, followed by castor oil when the fever rose, which operated favorably. On the next morning the gums were very slightly affected; perfectly free from fever —Sulp. Quina repeated—no return of fever.

*Case* 4. Nov. 12th 1828. On calling at the house of Mr. F. where I was attending on several patients ill of the fever, I met at the gate a negro boy for whom I had prescribed on the day before. Observing that he was very unwell, I directed him to lie down, and on coming in to prescribe for him, about an hour after, found him in a state of profound stupor, the pulse small, irregular, and too quick to be counted, the extremities cold, and the surface of the body rather warm, but dry, harsh and inelastic, the tongue covered with a secretion of white mucus—he was evidently in the stage of depression which precedes the fever. After a time the heat came to be generally diffused over the body, and was somewhat increased above the natural temperature. During the chill, the bowels were evacuated by stimulating enemata, and after the fever rose, very large sinapisms were applied over the stomach, to the extremities, and to the nape of the neck, and suffered to remain until some degree of permanent inflammation was produced, without producing any symptoms of returning sensibility

—indeed he was perfectly insensible to their action. Such was the prostration of the system that we were afraid to prescribe any thing of a debilitating nature. As the fever subsided, we gave him carb. of ammo. and infusion of serpentaria. At 9 o'clock P. M. the fever had so far subsided, that the skin was nearly of a natural temperature ; and on the next morning, the coma with the other unpleasant symptoms was relieved, but the debility was still very great. Calomel 15 grs. Dovers Powder 10 grs. divided into two powders, were taken in the course of the morning, followed by Ol. Ricini in the afternoon.

Slight fever on the preceding afternoon. The medicine had operated favorably ; all the symptoms much improved. Quinine, 8 grs. to be taken in the course of the morning. There was a slight return of fever for a few days, but the patient recovered his former health very rapidly.

It is in cases similar in character to those above described, that the plan of Dr. Daniel is peculiarly applicable. The rapid march of the disease does not afford time for slower vesicatories. Sinapisms produce their effect more speedily, may be applied to a much greater extent of surface, probably exert a greater influence over the heart and arteries, and are far less distressing to the patient. It will be observed that these unfavorable symptoms frequently depend upon temporary congestion, are very sudden in their accession, and when most alarming, frequently give way at the termination of the paroxysm. It is therefore an object of considerable importance to have the surface free for the application of the same remedial agent, upon the accession of another paroxysm.

*Case* 5. Nov. 15, 1823. In the absence of the family physician, (Dr. Rodgers,) I was called to visit a son of Mr. S. aged 11. He had had a chill in the morning followed by fever, which had nearly subsided when I saw him in the evening. This case exhibited no particular indications calculated to excite alarm—his skin was dry and husky but nearly of the natural temperature—the pulse not more hurried than is usual in the febrile diseases of children of that age—his respiration perfectly easy. If any symptom would have occasioned anxiety to the experienced physician, it would perhaps have been, the listlessness and perfect indifference which he manifested towards every thing around him. I gave him an emetic which operated imperfectly. On the next morning the family physician, without any suspicion of danger, prescribed a dose of calomel to be followed by some other cathartic, which was represented to have operated favorably.

On the 3d day of his illness he was surprised to find him at 10 A. M. with a chill, his extremities cold, his pulse sunk, and his countenance pale and ghastly. External warmth was applied, and an active course of stimulation adopted, consisting of the liberal administration of carb. of ammo. and the tinct. Opii.

Being requested to stay with him during the night, I saw him about 9 o'clock. The surface of the body was cold, except in the vicinity of the important viscera, where it was burning hot—the circulation in the extremities had ceased, while the action of the heart, as indicated by the pulsation of the carotids, was exceedingly rapid and violent. The administration of stimulants, as was to be expected, increased the action of the carotids without diffusing the excitement over the system—the respiration became more laborious, and at ten he expired.

*Case* 6. The daughter of Major R. aged 5 years, was taken with a chill, on the 12th of Nov. 1827. At the time I was staying at the house of her father, labouring under the sequelæ of bilious fever to a most distressing degree. After the chill, she lay very quietly, disposed to sleep, and complaining so little, that no serious apprehensions were excited. The fever returned on the next day about 12 o'clock, with some very alarming symptoms. The drowsiness was increased, the pulse small, quick and irregular, the tongue covered with a dark fur, and the general appearance of the patient appeared to indicate that the disease was about to assume an insidious congestive character. There was indistinct remission of the fever in the evening, with an improvement of many of the most unfavorable symptoms. A mercurial purgative, followed by repeated doses of castor oil, was given during the day, which operated very imperfectly.

On the next morning, (the 3d of her illness) at six o'clock, the chill returned with convulsions. Sinapisms and external heat were applied to the extremities, and in about two hours the chill terminated by a general diffusion of heat over the surface ; but the circulation on the surface was not restored, the skin retaining its pale and purple hue, the pulse in the radial artery was small and irregular—in the carotids excessively violent, great heat over the thorax and abdomen, with considerable fulness in the region of the liver. Active purgatives were again prescribed, which produced copious serous evacuations, slightly tinged with bile, with great irritation and distress. The fever remitted in the evening, without perspiration, and without relief of the coma. An anodyne enema was administered, and a blister applied over the hypochondriac and epigastric region.

On the next morning, the 4th of her illness, the patient tolerably clear of fever, stupor remaining. The fever returned again at 12 with an aggravation of the symptoms of congestion and oppression, a remission took place in the evening, followed by an exacerbation, at two o'clock on the morning of the 5th day, under which the patient sunk and died about 9 o'clock.

A very important difference will be remarked in the two last cases, and the preceding ones. It has always been a very serious source of regret to me that the prostration both of body and mind arising from the previous attack of fever, rendered me unable to give to the last patient that attention which the urgency of the symptoms demanded. At the time we were unacquainted with the great efficacy of external irritants in cases of this kind.—Perhaps in the last case, the lancet might have been useful, local bleeding would no doubt have been of service. Dr. Hamilton of Natches, informs me that in these prostrate cases of fever, he has found great advantage from a resort to the lancet, wherever the action of the heart and carotid arteries has been excessive.

*Case* 7. July 4 1828. I was called to see a negro boy 14 years of age, on the 3d day of his illness. He had been taken ill from home, and on the day before had been carried 30 miles in a gig. The stage of depression, indicated only by the coldness of the extremities and the weakness of his pulse, came on at 9 o'clock and in about an hour terminated in fever, with a general diffusion of heat. The pulse weak, irregular, rather full, from 110–120, respiration laborious, profound coma. The feet were immersed in hot water, and a vein opened in the arm, but when six ounces of blood had been drawn, the pulse began to sink the limbs became relaxed and a copious perspiration broke out from the forehead—the skin elsewhere remaining hot and dry as before. Calomel 15 grs. Tart of Antimony 1 gr. divided into 3 powders, one every 2 hours. As soon as leeches could be procured 30 were applied to the chest and epigastrium, and within half an hour after their application the patient was sufficiently relieved to ask for water. During the afternoon the operation of his medicine producing copious serous evacuations, was checked by an anodyne enema. In the course of the evening the fever subsided, the heat remaining in the thoracic and abdominal regions much longer than elsewhere, and in the mean time the pulse became fuller and more distinct. After the fever subsided a blister was applied to the epigastrium.

6th. Blister has drawn well. Calomel 15 grs. Dovers Powder 8 grs. to be taken in the morning, followed by an active cathartic in the afternoon.

7th. Clear from fever. Medicine operated well, bringing away copious tar-like evacuations. Quinine 12 grs. in the course of the morning. Slight return of fever for a few days, which yielded to mild cathartics.

This last was certainly a well marked case of congestive fever, the more deserving of notice, as it was accompanied with a general diffusion of heat over the surface of the body. It has been too much the practice of medical writers, to apply the term *congestive* to those cases of fever only, in which general reaction on the surface does not take place. This we think is an error. Although the action of the arterial and capillary system are very intimately connected, yet the latter is so far independent of the former, that reaction may take place on the surface, while the heart is oppressed with a load under which it can scarcely pulsate. Under the influence of this error, many writers have denied that increased heat is an invariable symptom of fever. Increased heat is so intimately associated with all *our* notions of fever, that we look upon it, almost, as the "morbus ipse." Diseases which prove to be febrile when permitted to run their course, may prostrate the system so suddenly, that fever is not developed. In the loose phraseology of popular conversation, it may be sufficient to say that "the patient died of malignant fever;" but popular error should not be permitted to conceal the important fact, that the *precursors of fever* constitute, in this case, the fatal disease.

One remark further on the operation of purgatives in this variety of fever, we deem it important to make. That is, their tendency to produce copious serous evacuations from the bowels, which debilitate the patient, without mitigating the violence of the disease. It is very necessary to attend to this in practice, and if on the one hand, we avoid drastic cathartics from a fear of increasing the debility, it is, on the other, equally important to avoid the other extreme of resorting to the saline medicines. Calomel guarded by opium, followed by mild but efficient purges, will secure all that is to be expected from this class of medicines, with less debility, than would result from any other course.

